# Recombinant Mal d 1 facilitates sublingual challenge tests of birch pollen‐allergic patients with apple allergy

**DOI:** 10.1111/all.12781

**Published:** 2015-11-06

**Authors:** T. Kinaciyan, B. Nagl, S. Faustmann, S. Kopp, M. Wolkersdorfer, B. Bohle

**Affiliations:** ^1^ Division of Immunology, Allergy and Infectious Diseases (DIAID)Department of DermatologyMedical University of ViennaViennaAustria; ^2^ Department of Pathophysiology and Allergy ResearchMedical University of ViennaViennaAustria; ^3^ Christian Doppler Laboratory for ImmunomodulationMedical University of ViennaViennaAustria; ^4^ Biomay AGViennaAustria; ^5^ Department of ProductionHospital PharmacySalzburgAustria

**Keywords:** Bet v 1, birch pollen‐related food allergy, Mal d 1, recombinant allergens

## Abstract

It is still unclear whether allergen‐specific immunotherapy (AIT) with birch pollen improves birch pollen‐related food allergy. One reason for this may be the lack of standardized tests to assess clinical reactions to birch pollen‐related foods, for example apple. We tested the applicability of recombinant (r) Mal d 1, the Bet v 1‐homolog in apple, for oral challenge tests. Increasing concentrations of rMal d 1 in 0.9% NaCl were sublingually administered to 72 birch pollen‐allergic patients with apple allergy. The dose of 1.6 μg induced oral allergy syndromes in 26.4%, 3.2 μg in 15.3%, 6.3 μg in 27.8%, 12.5 μg in 8.3%, 25 μg in 11.1%, and 50 μg in 4.2% of the patients. No severe reactions occurred. None of the patients reacted to 0.9% NaCl alone. Sublingual administration of 50 μg of rMal d 1 induced no reactions in three nonallergic individuals. Our approach allows straight forward, dose‐defined sublingual challenge tests in a high number of birch pollen‐allergic patients that *inter alia* can be applied to evaluate the therapeutic efficacy of birch pollen AIT on birch pollen‐related food allergy.

Birch pollen‐related food allergy (BPRFA) is the most prevalent food allergy in adolescent and adult individuals in regions where birch trees are indigenous 1. This IgE‐mediated food allergy is the consequence of initial sensitization to the major birch pollen allergen, Bet v 1, and subsequent immunological cross‐reactivity of Bet v 1‐specific IgE antibodies and T lymphocytes with structurally related proteins in diverse foods, most often apple, peach, and hazelnuts 2. Cross‐reactivity of Bet v 1‐specific IgE antibodies with homologous food allergens typically causes oral allergy syndromes (OAS), that is immediate reactions confined to the site of contact with fresh foods 3. Cross‐reactivity at the T‐cell level may induce visible late phase reactions, for example worsening of atopic eczema, even in the absence of OAS 4, 5.

More than 70% of birch pollen‐allergic patients develop allergic reactions to apple, and this perennial food allergy more drastically impairs their quality of life than the seasonal rhinoconjunctivitis 2. Currently, no effective treatment for BPRFA exists. Clinical and immunological evidence strongly support that Bet v 1 is the actual culprit in birch pollen‐related apple allergy. However, it is still under debate whether successful induction of clinical tolerance to Bet v 1 by allergen‐specific immunotherapy (AIT) with birch pollen concomitantly induces clinical tolerance to apple 3. Whereas some studies reported a reduction of apple‐induced symptoms during birch pollen AIT 6, 7, 8, 9, others found no general benefit for patients with improved pollinosis 10, 11, 12. Some patients even showed a worsening or the onset of BPRFA during AIT 10, 11, 12.

A major problem in evaluating the therapeutic efficacy of birch pollen AIT on the associated apple allergy is the current lack of standardized tests to assess food‐induced symptoms. In most studies, patients have been orally challenged with fresh apple before and after AIT. However, this approach cannot guarantee that the same doses of Mal d 1 are used at the different time points of challenge for several reasons. The concentration of Mal d 1 in apples is highly dependent on the cultivar and also influenced by storage conditions 13, 14. Moreover, Mal d 1 is easily destroyed during the preparation of challenge meals 15. Such problems might be overcome by the introduction of recombinant allergens for *in vivo* use. Indeed, we have previously reported that recombinant (r) Mal d 1 improved the sensitivity of skin prick testing in birch pollen‐allergic patients 16. In the present study, we tested the applicability of rMal d 1 for oral challenge tests of birch pollen‐allergic patients with concomitant apple allergy.

We included three nonallergic individuals and 72 Austrian birch pollen‐allergic patients (median age: 33.5 years; 37 female, 35 male) with a history of apple‐induced symptoms. The study was approved by the Ethics Committee of the Medical University of Vienna, and all patients gave written informed consent. Allergic patients displayed positive skin reactivity to birch pollen (ALK Abelló, Hørsholm, Denmark) and fresh apple (Pink Lady). Birch pollen‐specific IgE levels ranged from 1.4 to >100 kU_A_/l (median: 14.7 kU_A_/l), Bet v 1‐specific IgE levels from 1.5 to >100 kU_A_/l (median: 13.4 kU_A_/l), and Mal d 1‐specific IgE levels from 0.6 to >100 kU_A_/l (median: 5.4 kU_A_/l) as determined by ImmunoCAP (Thermo Fisher Scientific, Uppsala, Sweden). Mal d 1.0108‐GMP0902 (termed rMal d 1), the most abundant isoform of Mal d 1, was produced under good manufacturing practice (GMP) conditions by Biomay AG, Vienna, Austria, and its concentration, identity, stability, physico‐ and immunochemical properties, biological activity, purity as well as possible impurities and contaminants were analyzed using an appropriate set of analytical procedures. rMal d 1 fulfilled all requirements for quality documentation concerning biological investigational medicinal products in clinical trials (EMA/CHMP/BWP/534898/2008) and according to the note for guidance on specifications for test procedures and acceptance criteria for biotechnical/biotechnological products (CPMP/ICH/365/96) and was accepted by the Austrian regulatory authority ‘Austrian Federal Office for Safety in Health Care’ (Agentur für Gesundheit und Ernährungssicherheit GmbH (AGES) and Bundesamt für Sicherheit im Gesundheitswesen (BASG) Vienna, Austria). The challenge solution (75 μl of 0.9% NaCl containing no or the indicated concentrations of rMal d 1) was pipetted directly underneath the tongue, and the patients were asked not to swallow for at least 2 min. If no relevant objective symptoms such as erythema, blister formation, edema, and swelling of lips, tongue, and larynx occurred within 20 min, the next dose was administered until the maximum concentration of 50 μg of rMal d 1 was reached. All sublingual challenge tests were performed outside of the birch pollen season.

None of the 72 allergic individuals reacted to 0.9% NaCl alone. The first dose of 1.6 μg of rMal d 1 induced positive reactions in 19 of 72 (26.4%) of the studied individuals, 3.2 μg in 11 (15.3%), 6.3 μg in 19 (27.8%), 12.5 μg in 7 (8.3%), 25 μg in 8 (11.1%), and 50 μg in 3 (4.2%) patients. These data are depicted in Fig. 1. In total, 5 of 72 (6.9%) allergic patients did not react to the highest challenge dose representing a cumulative dose of 100 μg of rMal d 1. Sublingual administration of 50 μg of rMal d 1 induced no reaction in three nonallergic individuals. In summary, sublingual challenge with a cumulative dose of about 50 μg of rMal d 1 (48.6 μg) induced OAS in 89% of the 72 birch pollen‐allergic patients and rose to 93% by administration of another 50 μg of rMal d 1. No systemic reactions occurred. We have previously challenged another group of 20 birch pollen‐allergic patients with 50 μg of rMal d 1 on two consecutive days 17. This dose induced OAS in all patients on each day, and no systemic reactions were observed. Together, we conclude that sublingual administration of up to 50 μg of rMal d 1 is safe and will induce positive challenge tests in the majority of birch pollen‐allergic individuals with associated apple allergy.

**Figure 1:**
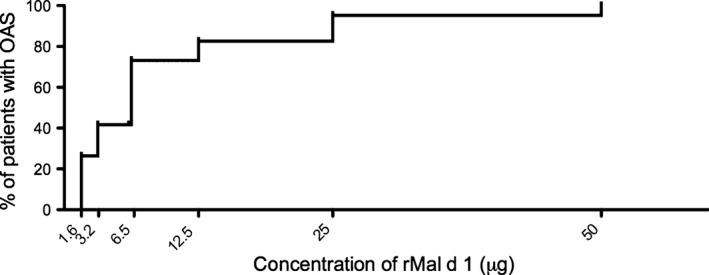
Sublingual challenge tests with increasing doses of rMal d 1. The percentage of 72 birch pollen‐allergic patients developing oral allergy syndromes (OAS) upon sublingual challenge with indicated doses of rMal d 1 is shown; challenges were stopped when OAS occurred.

Our proof‐of‐concept study demonstrates that sublingual challenges with rMal d 1 work and can be performed in a simple and fast manner. Defined doses of the recombinant protein can be directly administered to the patient. No preparation of challenge meals is necessary. Another great advantage of this straight forward approach is the fact that challenges with rMal d 1 can be equally performed in different allergy centers at different time points. By this, it will be feasible to evaluate BPRFA before and during the course of AIT with birch pollen in large patient cohorts. Such evaluations will help to clarify whether AIT with inhalant allergens benefits the associated food allergy.

This proof‐of‐concept study employed a food allergen known to induce mild allergic symptoms. However, the concept presented here can be expanded to the use of allergens causing more severe, systemic reactions, such as Gly m 4 and the Bet v 1‐homolog in soy 18, and thereby improve proper diagnosis of potentially life‐threatening food allergy 19. Along these lines, sublingual challenges with recombinant allergens may be applied to define threshold levels of food‐induced reactions and thereby contribute to the development of reference doses and action levels for allergens in foods 20.

The current EAACI food allergy and anaphylaxis guidelines recommend to advise food‐allergic patients to avoid the culprit foods 19. However, dietary restrictions impair the quality of life of the affected persons. Therefore, an elimination diet should be recommended only if food allergy is based on a clear history or observed after oral provocation tests 3. In cases of inconclusive history, oral challenge tests with recombinant allergens might be easily performed in the doctor′s practice. Solved in tasteless and colorless solutions, recombinant allergens will also facilitate double‐blind placebo‐controlled food challenges. Thus, the use of recombinant food allergens may revolutionize accurate diagnosis of food allergy in the future.

## Conflict of interest

The authors declare no competing financial interests.
